# A Phylogenetic Perspective on Biogeographical Divergence of the Flora in Yunnan, Southwestern China

**DOI:** 10.1038/srep43032

**Published:** 2017-02-21

**Authors:** Shuiyin Liu, Hua Zhu, Jie Yang

**Affiliations:** 1 Center for Integrative Conservation, Xishuangbanna Tropical Botanical Garden, Chinese Academy of Sciences, Kunming, China; 2 University of Chinese Academy of Sciences, Beijing, China; 3 Key Laboratory of Tropical Forest Ecology, Xishuangbanna Tropical Botanical Garden, Chinese Academy of Sciences, Kunming, China

## Abstract

In recent years, an increasing number of studies incorporated biogeography with phylogenetic analyses to reveal the origin and evolutionary history of specific floras. In this study, we constructed the mega-phylogeny of the floras of three representative regions across Yunnan, southwestern China. We analyzed the phylogenetic structure and beta diversity based on the presence/absence of species (genus or family) data to investigate the phylogenetic patterns of regional floras. We found conspicuous divergence at the genus and species level in the pattern of phylogenetic structures, which most likely related to historical biogeography. The flora of southern Yunnan was shaped by the strike-slip extrusion of Indochina and the regional climatic stability, while the flora of northwestern Yunnan was shaped by the uplift of the Himalaya-Tibetan Plateau and the oscillations of the glacial-interglacial periods. The flora of central Yunnan had nearly equal proportions of the northern and southern floras that may be derived from a common Tertiary tropical or subtropical flora. Geological events fit well with the floristic and phylogenetic patterns across Yunnan. This study highlighted the importance of linking phylogenetic analyses to biogeographic interpretations to improve our understanding of the origin, evolution and divergence of regional floras.

It is widely accepted that geological history, evolutionary events and processes, and current climatic conditions are significant factors in the formation of contemporary biogeographic patterns^1^,^2^. A time-calibrated phylogeny contains information about the phylogenetic relationships among species and the timing of evolutionary processes, such as speciation, divergence, and extinction, and can therefore be used to extract information about the evolutionary history of biological communities or floras at different temporal and spatial scales^3^. Recently, the term ‘phylofloristics’ was introduced to describe an analytical approach that relates the compositional similarity between entire floras to spatial and environmental distances in order to phylogenetically investigate a floristic assembly^4^. Phylogenetic measures provide an estimate of how much of the evolutionary history is represented in a particular region, providing a novel research and analytical approach for biogeography and making it possible to infer the relative influence of various biogeographical events in the historical period on the phylogenetic patterns of floras^4^,^5^,^6^. In this study, we applied this concept to investigate the contemporary pattern of floras and the associated geological events during the collision between India and Eurasia.

The collision between India and Eurasia, which began in the early Cenozoic, may be the largest active orogenic event on earth^7^,^8^. This long-lasting and ongoing tectonic process triggered associated geological events ranging from the uplift of the Himalaya-Tibetan plateau to the lateral extrusion of the continental landmass^9^,^10^. The associated orogenic and environmental effects (e.g., geomorphology and climate change), especially the later large-scale uplifts on or around the Himalaya-Tibetan Plateau^11^ and the strike-slip extrusion of Indochina, were reported to have driven and influenced genetic discontinuities, speciation, and the evolution of numerous plant and animal groups, as well as generating the contemporary biotic distribution pattern^7^,^12^,^13^. The species-based study on the biogeography of southern, central and northwestern Yunnan found that these three floras might be derived from a common flora, but the geological history of each region has influenced its flora, and they have remained divergent since the late Tertiary^14^,^15^,^16^. However, this biogeographical hypothesis lacks the support of phylogenetic evidence at a regional scale. Phylofloristics give us new insight to use phylogenetic information to demonstrate that the multistage uplift of the Himalayas and the strike-slip extrusion of Indochina resulted in the contemporary patterns of biogeography or initiated the biogeographical divergence of floras at a regional scale.

In this study, we investigated the floristic phylogenetic patterns of three representative regions in southern, central, and northwestern Yunnan. The present study aimed to examine the significance of key geological events, particularly the plate collision-induced uplift of the Himalaya-Tibetan Plateau and the strike-slip extrusion of Indochina, on contemporary patterns of biogeography using a comprehensive method combining floristic geography with phylogenetic information. Several factors made these regions ideal for this research. First, the Yunnan region is located at a sutural zone between Laurasia and Gondwana in geological history^17^,^18^, and an evident biogeographical divergence is believed to have occurred between these three regional floristic compositions in response to the uplift of Himalaya and extrusion of the Indochina block^14^,^15^,^16^. Second, floristic inventories of angiosperms in these three areas are well documented^15^,^19^,^20^,^21^. Third, the biogeography and divergence of these three floras were investigated on the basis of analyses of floristic geography^14^,^15^,^16^ without incorporating the phylogenetic patterns of these floras, which would allow the deduction of the influence of geological history on the floristic assembly with an evolutionary perspective.

We constructed mega-phylogenies of the regional floras and then analyzed the phylogenetic assemblage structure in each flora, as well as the phylogenetic beta diversity between floras from three taxonomical hierarchies (family, genus and species). These phylogenetic analyses were also conducted among different geographical elements (e.g., pantropic or tropical Asia genus, north temperate or temperate Asia genus) for more detailed phylogenetic investigations of these floras. Specifically, we addressed the following questions: (i) What is the pattern of the phylogenetic structure in three floras across Yunnan (i.e., southern, central and northwestern Yunnan)? (ii) What is the phylogenetic beta diversity between these floras? and (iii) Do the results integrated with phylogenetic information agree with previous biogeographic hypotheses in terms of the biogeographical divergence of the flora across Yunnan initiated by the uplift of Himalaya and extrusion of Indochina? Based on the biogeographic history described above and predicted patterns for community phylogenetic structure, we proposed three hypotheses. (i) The assemblages of flora in northwestern Yunnan are clustered, especially to temperate floristic elements, due to the quick speciation and divergence of abundant closely related taxa with the uplift of Himalaya. (ii) In contrast, the southern flora is more phylogenetically overdispersed, especially to tropical floristic elements, in response to the collision between India and Asia. This geological event resulted in southern Yunnan as a sutural zone, where distantly related taxa from Indo-Malesian flora (from Gondwana) and East Asia flora (from Laurasian) came together. (iii) The phylogenetic similarity between central and northwestern flora is nearly identical to the similarity between central and southern flora, which could derive from a common flora in geological time.

## Results

### Patterns of phylogenetic structure

**Figure 1:**
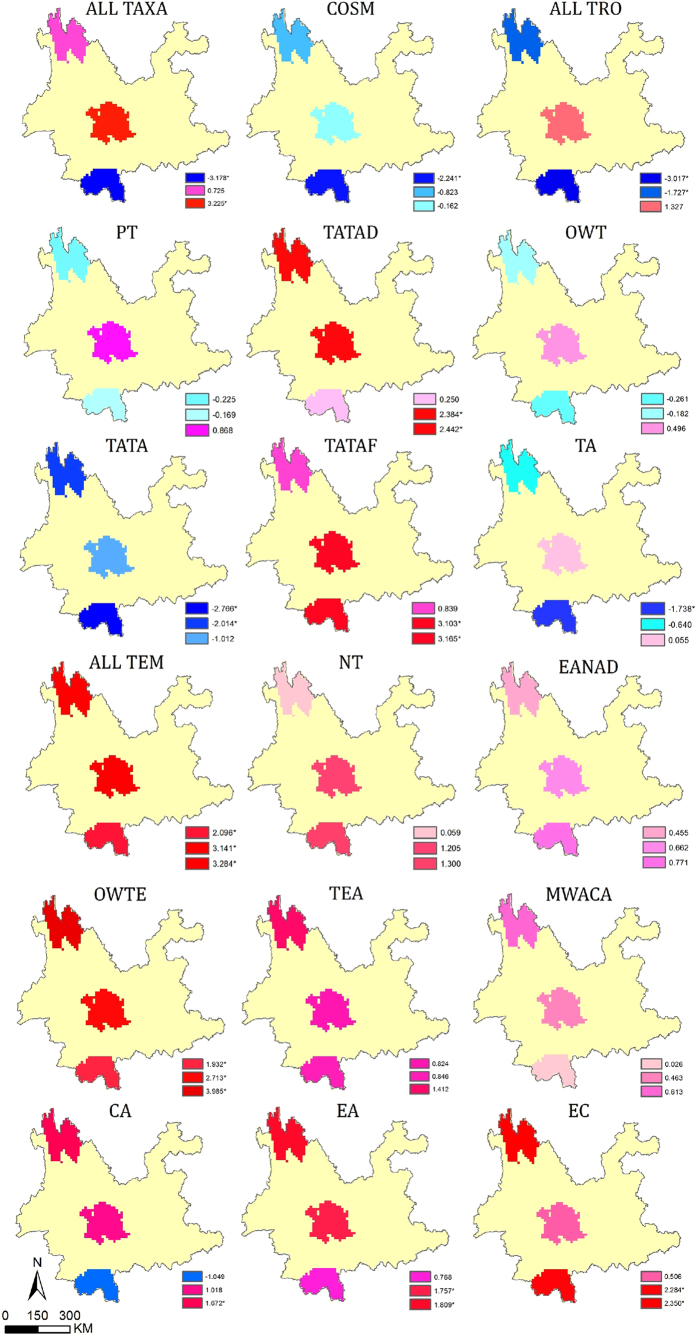
The net relatedness index (*NRI*) of the three floras at the genus level. *The *p*-values corresponding to *NRI* less than 0.05. ALL TAXA, all taxa at the generic level; COSM, cosmopolitan; ALL TRO, all taxa with tropical distributions; PT, pantropic; TATAD, tropical Asia and tropical America disjointed; OWT, old world tropic; TATA, tropical Asia to tropical Australia; TATAF, tropical Asia to tropical Africa; TA, tropical Asia; ALL TEM, all taxa with temperate distributions; NT, north temperate; EANAD, east Asia and North America disjointed; OWTE, old world temperate; TEA, temperate Asia; MWACA, Mediterranean, west Asia to center Asia; CA, center Asia; EA, east Asia; and EC, endemic to China. The map was generated using ArcGIS 10.2.2. (http://www.esri.com).

**Figure 2:**
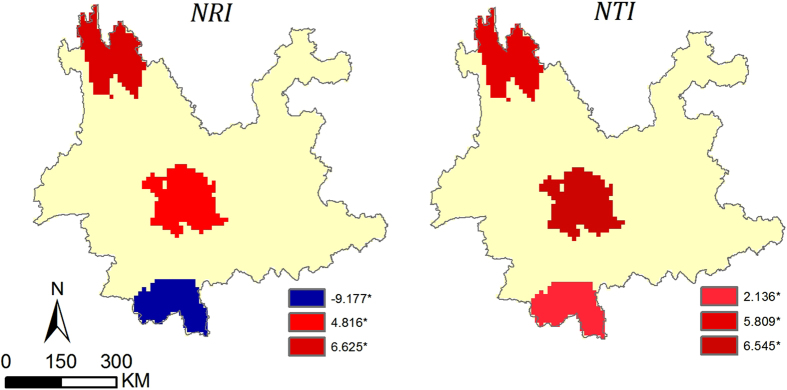
Phylogenetic structure of the three floras at the species level. *The *p*-values corresponding to *NRI* or *NTI* less than 0.05. *NRI*, the net relatedness index and *NTI*, the nearest taxon index. The map was generated using ArcGIS 10.2.2. (http://www.esri.com).

**Figure 3:**
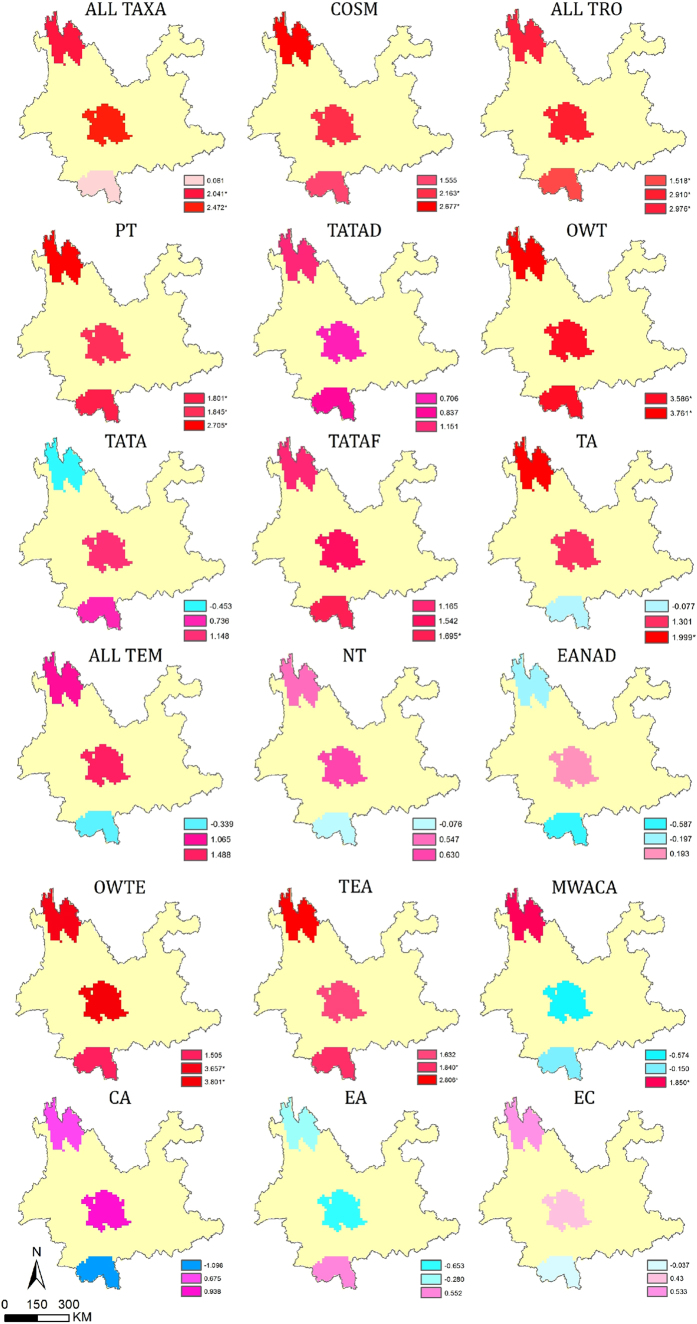
The nearest taxon index (*NTI*) of the three floras at the genus level. *The *p*-values corresponding to *NTI* less than 0.05. The map was generated using ArcGIS 10.2.2. (http://www.esri.com).

At the family level, the patterns of phylogenetic structure in these three regions were less divergent (Figures S1 and S2) but distinctly differed at the genus and species level. The genera and species of southern Yunnan exhibited a significant pattern of phylogenetic overdispersion across the entire tree (*NRI*), while genera and species in central and northwestern Yunnan were clustered (Fig. 1, ALL TAXA and Fig. 2). Genera that had a tropical distribution in southern Yunnan, which made up the majority of the genera (76.7% of the total), also showed significant overdispersion, particularly those with tropical Asia and tropical Asia to tropical Australia distributions. Interestingly, this pattern of overdispersion was also seen in the northwestern genera with a tropical distribution, which made up 43.5% of all genera (Fig. 1, ALL TRO-TA). However, northwestern genera with temperate distributions (45.8% of all genera) were highly clustered, especially those with old world temperate, temperate Asia and center Asia distributions (Figs 1 and 3, ALL TEM-CA). Moreover, with respect to the terminals (more recent divergences), the species and genera with a tropical distribution in southern Yunnan were clustered (Fig. 3, ALL TRO-TA and Fig. 2, *NTI*).

The *p*-values of all *NRI* and *NTI* measures and the taxonomic richness of these groups are available in the Supplementary Material (Table S1).

### Phylogenetic beta diversity

The floras of the three regions at the family level were very similar (over 0.837), followed by those at the genus level (0.624–0.747), but fairly dissimilar at the species level (0.324–0.530) (Table 1). The phylogenetic similarities between the floras of central and southern Yunnan were similar to those between central and northwestern Yunnan at all taxonomic levels and geographic elements, implying that central Yunnan had nearly equal proportions of the southern and northwestern floras. Moreover, we found that the phylogenetic similarity scores of families or genera with tropical distributions were both notably higher than between temperate families or genera, which indicated a close phylogenetic relationship among tropical elements of these three floras. Additionally, higher positive values of *S.E.S. D*_*nn*_ in the tropical families and genera than temperate families and genera suggested that tropical and temperate taxa had smaller phylogenetic beta diversity than expected, and tropical taxa had a lower diversity than temperate taxa among these regions (Table 2).

## Discussion

In this study, we extended traditional species-centric floristic analyses and phylogenetic analyses of certain taxa or local floras to incorporate phylogenetic information and focus on regional floras. We used presence/absence floristic data of regions in southern, central, and northwestern Yunnan to analyze the phylogenetic structure and beta diversity of the three regional floras to quantify how closely related pairs of taxa were within or between floras. Our results showed a conspicuous divergence at the genus and species level in the pattern of phylogenetic structures among the three representative floras. We integrated these results with the influence of geological historical events on the biogeographical divergence of regional floras to assist with interpretation.

The pattern of phylogenetic structure in the flora of southern Yunnan was overdispersed. The assemblages of species (or genera) in southern Yunnan were more phylogenetically dispersed across the whole phylogeny (*NRI*), and the same was found for the tropical genera, especially with tropical Asia and tropical Asia to tropical Australia distributions (Fig. 1, ALL TAXA, ALL TRO, TATA, TA and Fig. 2, *NRI*). This pattern aligned with the geological and biogeological events, which was the collision between India and Asia that displaced Indochina southeastward along the Ailao Shan-Red River shear zone^7^,^22^. The land and tropical climate of southern Yunnan, located at a sutural zone between Gondwana and Laurasia^17^,^18^, were shaped during this geological event. Thus, the southern biome was conspicuously linked to the dispersal of distantly related species from Palaeotropic regions and South China. Our phylogenetic results illustrated that geological changes and the associated dispersal occurred in this region. This was generally compatible with the biogeographical hypothesis, which proposed that the flora of southern Yunnan evolved in concert with the extrusion of the Indochina block and was mainly influenced by tropical Asian elements since the later Tertiary^16^.

However, the *NTI* values indicated that, with respect to the more recent divergences, the species and tropical genera in southern Yunnan were clustered (Fig. 3, ALL TRO-TA and Fig. 2, *NTI*). This pattern may relate to the evolution and reproduction of species under a stable tropical climate. Phylogenetic clustering at a regional scale may result from the rapid speciation rates or slow extinction rates in evolutionary time^23^,^24^. Tropical regions were expected to have relatively low rates of extinction and high rates of speciation^14^,^25^,^26^. The different results of *NRI* and *NTI* were due to the different identification and measurements, which emphasized the importance of the simultaneous use of two indices providing diverse information from the whole phylogeny and the more recent divergences.

The floristic composition of northwestern Yunnan showed significant phylogenetic clustering. The species (or genera) in northwestern Yunnan were clustered across the entire phylogeny (*NRI*) and with respect to the more recent divergences (*NTI*), and the same was found for the temperate genera (Fig. 1, ALL TAXA, ALL TEM; Figs 2 and 3, ALL TAXA and ALL TEM). This pattern indicated the coexistence of substantial closely related species. This clustering was consistent with the biogeographical hypothesis, as the flora of northwestern Yunnan evolved with the uplift of the Himalayas and by the gradual proliferation of mainly cosmopolitan and north temperate floristic elements^16^. The plate collision-induced uplifting of the Himalayas-Tibetan Plateau began in the early Cenozoic and was relatively rapid and uniform, particularly during the Quaternary period^27^,^28^. Taxa on the Himalaya-Tibetan Plateau became isolated from peripheral regions with the appearance of massive mountains and deep valleys. The continued uplift finally cut off the genetic exchange between the Himalayan range and the interior Tibetan Plateau^7^, promoting local speciation and hybridization of many closely related species, such as those in the genera *Saussurea*^12^, *Pedicularis*^13^, and *Meconopsis*^29^. The patterns of phylogenetic structure we observed in northwestern Yunnan reflected these geological and evolutionary processes.

We also observed that tropical genera of northwestern Yunnan, particularly those with tropical Asia and tropical Asia to tropical Australia distributions, were phylogenetically overdispersed across the whole phylogeny (*NRI*) (Fig. 1, ALL TRO, TATA, TA). The severe environments during the Quaternary may have filtered out many closely related tropical elements, leading to this overdispersed pattern. Another reason for such a pattern was that northwestern Yunnan may have acted as a refuge during glacial periods by providing diverse habitats in low, warm areas for plants distantly related to the local flora. This was consistent with an investigation of the Qinghai-Tibetan Plateau^30^, which detected phylogenetic overdispersion at the junction of the eastern edge of the plateau due to shelters during glacial periods.

Lower phylogenetic beta diversity and closer phylogenetic relationships existed among the tropical elements in these three floras rather than among the temperate elements (Tables 1 and 2). In addition, the flora of central Yunnan had nearly equal proportions of the northern and southern floras, and the phylogenetic similarities of families among the three regions were very high, which may due to the possible common origin. However, the different geological and evolutionary histories after the Tertiary were most likely to have driven the biogeographical divergence, which explained the relatively low similarity of species in focused areas. The uplift of the mountains and the oscillations in the climate created severe living conditions during the Quaternary^31^ that acted as a barrier to dispersal, making it difficult for species from the lowlands to migrate to the northern area. Furthermore, niche conservatism suggested that few clades crossed ecophysiological barriers to harsher environments^32^,^33^ due to the conserved ecological traits^34^,^35^,^36^. This suggests that various tropical elements were present in northwestern Yunnan before the Quaternary period, which might explain such a close relationship among tropical elements. These results also supported the hypothesis that the floras of Yunnan were derived from a common Tertiary flora distributed in tropical or subtropical Asia^16^.

Taken together, the integration of floristic geography with phylogenetic information in our analyses provided clues that revealed the influence of geological events on the biogeographical divergence of regional floras, as well as evidences for biogeographical hypotheses about Yunnan. The uplift of the Himalayas and the extrusion of Indochina fit well with the phylogenetic patterns of regional floras across Yunnan. However, our results were no doubt coarser than would be found with a more refined supertree based on DNA sequences. The method used to build the phylogeny in this study was actually not ideal, especially when some genera or species were missing in the PhytoPhylo mega-phylogeny. In addition, we should be cautious about the limitations of using phylogenetic trees to date the timing of evolutionary processes because of the crucial role of fossils, especially when using these times to interpret the biological impacts of geological events^37^,^38^. Thus, it is essential to improve the method of constructing a robust supertree, the ability of acquiring considerable DNA sequences and the study of plant fossils, which will help us reveal the origin, evolution and divergence of regional floras more objectively and truly.

## Methods

### Study area

**Figure 4:**
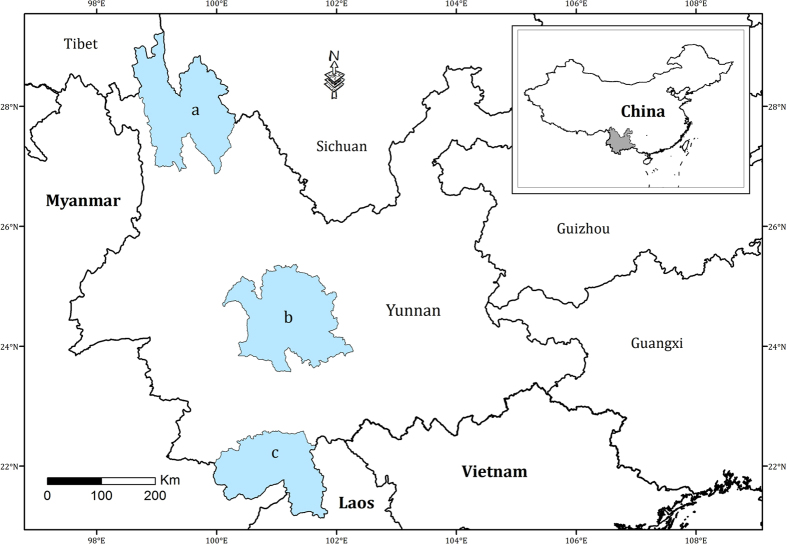
Location of the study regions. (**a**) Northwestern Yunnan; (**b**) central Yunnan; and (**c**) southern Yunnan. The map was generated using ArcGIS 10.2.2. (http://www.esri.com).

Yunnan province is located in southwestern China, between 21°09′–29°15′N and 97°32′–106°12′E. The present analyses utilized the floras of three representative regions in southern, central and northwestern Yunnan (Fig. 4). Southern Yunnan (21°09′–22°36′N and 99°58′–101°50′E), which includes Xishuangbanna Dai Autonomous Prefecture, has a tropical monsoon climate and a low mountain-basin topography, with altitudes ranging from 475 m to 2,430 m across an area of 19,690 km^2^. Its lowlands are often dominated by tropical rainforest^39^. Central Yunnan (23°53′–25°11′N and 100°32′–101°58′E), which includes 7 counties and incorporates the core regions of Mt. Wuliangshan and Mt. Ailaoshan, has a subtropical climate and a middle mountain-valley topography, with altitudes ranging from 422 m to 3,157 m (mainly 1,300 m to 2,200 m) across an area of 25,424 km^2^. It is mostly covered by subtropical, evergreen broad-leaf forests and secondary *Pinus yunnanensis* forests. Northwestern Yunnan (27°10′–28°27′N and 98°53′–99°42′E), which includes 3 counties located at the center of the Hengduan Mountains, has a temperate climate and an alpine-deep valley topography, with altitudes ranging from 1,900 m to 6,740 m across an area of 23,870 km^2^. Its main vegetation consists of temperate sclerophyllous oak forests and cold temperate coniferous forests^16^.

### Floristic data

Comprehensive synonymized inventories of the total angiosperms in southern, central and northwestern Yunnan were obtained based on checklists from several monographs^15^,^19^,^20^,^21^, the Flora of Yunnan^40^ and a database of angiosperms from the herbarium of the Kunming Institute of Botany, Chinese Academy of Sciences. Only native species were used in the study. All intraspecific taxa (e.g., subspecies, variety and forma) were not included in the analyses. A total of 9,370 angiosperm species from 1,860 genera and 212 families were used in this study. We checked and standardized the spelling and nomenclature of species in the floras according to The Plant List version 1.1 (TPL, available at http://www.theplantlist.org), which is an international standard database for plant nomenclature. This process aimed to maximize the match between names in the local lists and those in the backbone phylogenetic hypothesis for construction of the phylogeny. All the names in the species list that were considered synonyms were replaced with their accepted names from the TPL. We assigned each species to a family using the R package ‘plantlist’^41^,^42^ following the TPL database, in which the circumscription of angiosperm families was generally consistent with APG III^43^.

Furthermore, to explore the phylogenetic information concerning taxa with different patterns of geographical distribution in the study areas, patterns of angiosperms distribution were quantified at the genus and family levels based on Wu^44^ and Wu *et al*.^45^,^46^. The distribution patterns of 6 families and 5 genera were unclear, so we excluded these taxa from thephylogenetic analysis of geographical elements. Across the three regions, a total of 13 types of geographical distribution at the family level and 15 types of geographical distribution at the generic level were included.

We built a presence/absence matrix at each level (i.e., species, genus and family) based on the presence or absence of each angiosperm in each region for the following calculation of phylogenetic structure and beta diversity.

### Mega-phylogeny construction

We constructed phylogenies by grafting the families, genera and species included in the study onto a backbone phylogenetic hypothesis in the R package ‘S. PhyloMaker’^47^. The backbone of this supertree was the PhytoPhylo mega-phylogeny^47^, an updated and expanded version of Zanne *et al*.’s species-level phylogeny^33^, which included 30,771 seed plants and was time-calibrated for all branches using seven gene regions available in GenBankand fossil data. PhytoPhylo included all families of extant seed plants in the world^48^ and had five times more genera and over 55 times more species than the newest angiosperm supertrees (i.e., R20120829)^47^. Genera and species that were not found in the PhytoPhylo mega-phylogeny were handled by S. PhyloMaker in one of three ways: (1) adding genera or species as polytomies within their families or genera; (2) randomly adding genera or species within their families or genera; and (3) adding genera or species to their families or genera with the same approach used in the online software Phylomatic and BLADJ. Using these three approaches, three phylogenies were generated at each level of resolution (i.e., family, genus and species). The phylogenies with the first approach are available in the Supplementary Material (Figures S3, S4 and S5). However, we mainly reported results based on the first approach. The detailed results using the other approaches are available in the Supplementary Material (Tables S2 and S3).

### Phylogenetic structure

The phylogenetic structure of each flora was quantified using two indices: the net relatedness index (*NRI*) and the nearest taxon index (*NTI*). In general, *NRI* is considered more sensitive to tree-wide patterns of phylogenetic clustering and overdispersion, while *NTI* is more sensitive to patterns of overdispersion and clustering closer to the tips of the phylogenetic tree^49^. *NRI* and *NTI* are defined as the measure of standardized effect size of mean pairwise distance (*MPD*) and mean nearest taxon distance (*MNTD*), respectively. These indices describe the difference in phylogenetic distance (i.e., *MPD* or *MNTD*) between observed and null communities generated with some randomization methods divided by the standard deviation of phylogenetic distance in the null communities^50^. They are computed as follows:


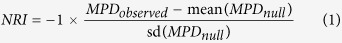



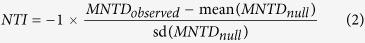


where *MPD*_*observed*_ (or *MNTD*_*observed*_) is the observed *MPD* (or *MNTD*), mean (*MPD*_*null*_) [or mean (*MNTD*_*null*_)] is the average *MPD* (or *MNTD*) of randomly generated assemblages, and sd (*MPD*_*null*_) [or sd (*MNTD*_*null*_)] is the standard deviation of *MPD* (or *MNTD*) in the null assemblages. We randomly shuffled the tip labels of the constructed phylogeny 999 times to generate null communities. A positive *NRI* (or *NTI*) value indicated a smaller phylogenetic distance among co-occurring taxa than expected (i.e., phylogenetic clustering). Conversely, a negative *NRI* (or *NTI*) value indicated a greater phylogenetic distance among co-occurring taxa than expected (i.e., phylogenetic overdispersion).

In this study, we used all the species in the three floras as a species pool and each study area as a plant community at the regional scale.

### Phylogenetic beta diversity

Phylogenetic beta diversity provides insights into the phylogenetic relationships between communities. Previous studies demonstrated that *D*_*pw*_ (mean pairwise phylogenetic distance) was unsuitable for large-scale studies, because it did not vary significantly along environmental and distance gradients^51^. Thus, the present analyses used *PhyloSor* (phylogenetic Sørensen index) and *D*_*nn*_ (nearest neighbor phylogenetic distance) to evaluate the phylogenetic relationships between floras. *PhyloSor* is defined as the ratio of branch length between shared taxa to the total branch length of all taxa in two ecological communities^52^. A larger *PhyloSor* value indicates closer community relationships^51^. *D*_*nn*_ is defined as the mean phylogenetic distance between a species in community *A* and its most-related species in community *B*. We used a null model analysis to test whether regional floras were more or less phylogenetically similar than expected by chance^53^. Negative values indicated a higher than expected phylogenetic distance, and positive values indicated a lower than expected phylogenetic distance. Specifically, the *PhyloSor* and the standardized effect size (*S.E.S*.) of *D*_*nn*_ can be quantified as follows:


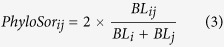



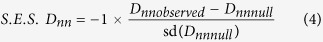


where *BL*_*ij*_ is the branch length between taxa shared by two communities, and *BL*_*i*_ (or *BL*_*j*_) is the branch length between all taxa of community *i* (or *j*). *D*_*nnobserved*_ is the observed dissimilarity between floras. *D*_*nnnull*_ is the mean of *D*_*nn*_ of the null distributions generated by randomly shuffling the tip labels of the phylogeny 999 times. sd (*D*_*nnnull*_) is the standard deviation of *D*_*nn*_ in the null distributions.

Analyses of phylogenetic structure and beta diversity were performed using the R packages ‘picante’^49^ and ‘vegan’^54^. The results of the phylogenetic structure were mapped with ArcGIS 10.2.2.

## Additional Information

**How to cite this article:** Liu, S. *et al*. A Phylogenetic Perspective on Biogeographical Divergence of the Flora in Yunnan, Southwestern China. *Sci. Rep.* **7**, 43032; doi: 10.1038/srep43032 (2017).

**Publisher's note:** Springer Nature remains neutral with regard to jurisdictional claims in published maps and institutional affiliations.

## Supplementary Material

Supplementary Information

## Figures and Tables

**Table 1:** Phylogenetic similarity (*PhyloSor*) among the three floras across Yunnan.

Groups	At the family level			At the genus level			At the species level		
	BN-ZB	ZB-DQ	DQ-BN	BN-ZB	ZB-DQ	DQ-BN	BN-ZB	ZB-DQ	DQ-BN
All taxa	0.904	0.906	0.837	0.747	0.744	0.624	0.530	0.453	0.324
Cosmopolitan	0.944	0.976	0.955	0.872	0.862	0.821	—	—	—
tropical distributions	0.918	0.888	0.828	0.754	0.794	0.670	—	—	—
PT	0.948	0.876	0.852	0.828	0.855	0.777	—	—	—
TATAD	0.925	0.939	0.867	0.793	0.868	0.766	—	—	—
OWT	0.913	1.000	0.913	0.827	0.792	0.758	—	—	—
TATA	0.923	1.000	0.923	0.758	0.789	0.682	—	—	—
TATAF	0.614	—	—	0.755	0.836	0.688	—	—	—
TA	0.766	0.827	0.609	0.677	0.722	0.558	—	—	—
Temperate distributions	0.880	0.903	0.813	0.743	0.719	0.577	—	—	—
NT	0.899	0.962	0.861	0.731	0.800	0.605	—	—	—
EANAD	0.880	0.885	0.851	0.804	0.672	0.613	—	—	—
10. OWTE	—	—	—	0.687	0.657	0.527	—	—	—
TEA	—	—	—	0.814	0.497	0.405	—	—	—
MWACA	—	—	—	0.870	0.763	0.644	—	—	—
CA	—	—	—	0.380	0.357	0.392	—	—	—
EA	0.841	0.801	0.653	0.696	0.758	0.552	—	—	—
EC	1.000	0.437	0.437	0.543	0.415	0.265	—	—	—
ESHD	—	—	—	—	—	—	—	—	—

BN, southern Yunnan; ZB, central Yunnan; and DQ, northwestern Yunnan.

**Table 2:** The standardized effect sizes of phylogenetic beta diversity (*S.E.S. D*_*nn*_) among the three floras across Yunnan.

Groups	At the family level			At the genus level			At the species level		
	BN-ZB	ZB-DQ	DQ-BN	BN-ZB	ZB-DQ	DQ-BN	BN-ZB	ZB-DQ	DQ-BN
All taxa	137.34	111.39	154.30	235.49	239.39	347.22	304.50	410.84	589.18
Cosmopolitan	26.82	27.82	27.00	28.23	30.62	30.63	—	—	—
tropical distributions	41.12	35.04	37.58	128.33	99.27	117.83	—	—	—
PT	33.16	28.45	29.60	54.69	52.65	54.37	—	—	—
TATAD	10.68	10.72	10.33	18.08	18.73	17.14	—	—	—
OWT	5.17	5.18	5.17	36.89	31.16	32.62	—	—	—
TATA	6.30	6.43	6.30	36.00	30.76	31.72	—	—	—
TATAF	0.08	—	—	27.36	25.31	25.63	—	—	—
TA	6.69	4.99	4.57	55.33	42.91	46.79	—	—	—
Temperate distributions	18.88	20.48	19.04	66.24	86.81	70.83	—	—	—
NT	14.83	16.61	14.94	34.73	48.26	36.51	—	—	—
EANAD	8.51	8.49	8.57	20.03	22.50	19.52	—	—	—
OWTE	—	—	—	20.48	26.32	20.26	—	—	—
TEA	—	—	—	6.92	6.96	6.04	—	—	—
MWACA	—	—	—	7.34	10.76	7.08	—	—	—
CA	—	—	—	0.70	3.60	2.47	—	—	—
EA	3.76	5.24	3.50	30.20	40.41	29.95	—	—	—
EC	—	0.33	0.33	8.67	11.03	8.07	—	—	—
ESHD	—	—	—	—	—	—	—	—	—

BN, southern Yunnan; ZB, central Yunnan; and DQ, northwestern Yunnan.
