# Supplementary material for: A Phylogenetic Perspective on Biogeographical Divergence of the Flora in Yunnan, Southwestern China
Source: Sci Rep. 2017 Feb 21;7:43032. doi: 10.1038/srep43032 (PMC5318862; doi:10.1038/srep43032)
Supplement: Supplementary Information [file srep43032-s1.pdf]

A Phylogenetic Perspective on Biogeographical Divergence of the Flora in Yunnan, Southwestern China

Shuiyin Liu[1,3], Hua Zhu[1*], Jie Yang[2*]

[1]Center for Integrative Conservation, Xishuangbanna Tropical Botanical Garden, Chinese Academy of Sciences, Kunming, China, [2]Key Laboratory of Tropical Forest Ecology, Xishuangbanna Tropical Botanical Garden, Chinese Academy of Sciences, Kunming, China, [3]University of Chinese Academy of Sciences, Beijing, China

* Correspondence and requests for materials should be addressed to H.Z. (zhuh@xtbg.ac.cn) and J.Y. (yangjie@xtbg.org.cn)

Figures

Figure S1. The net relatedness index (*NRI*) of the three floras at the family level. The map was generated using ArcGIS 10.2.2. (http://www.esri.com)

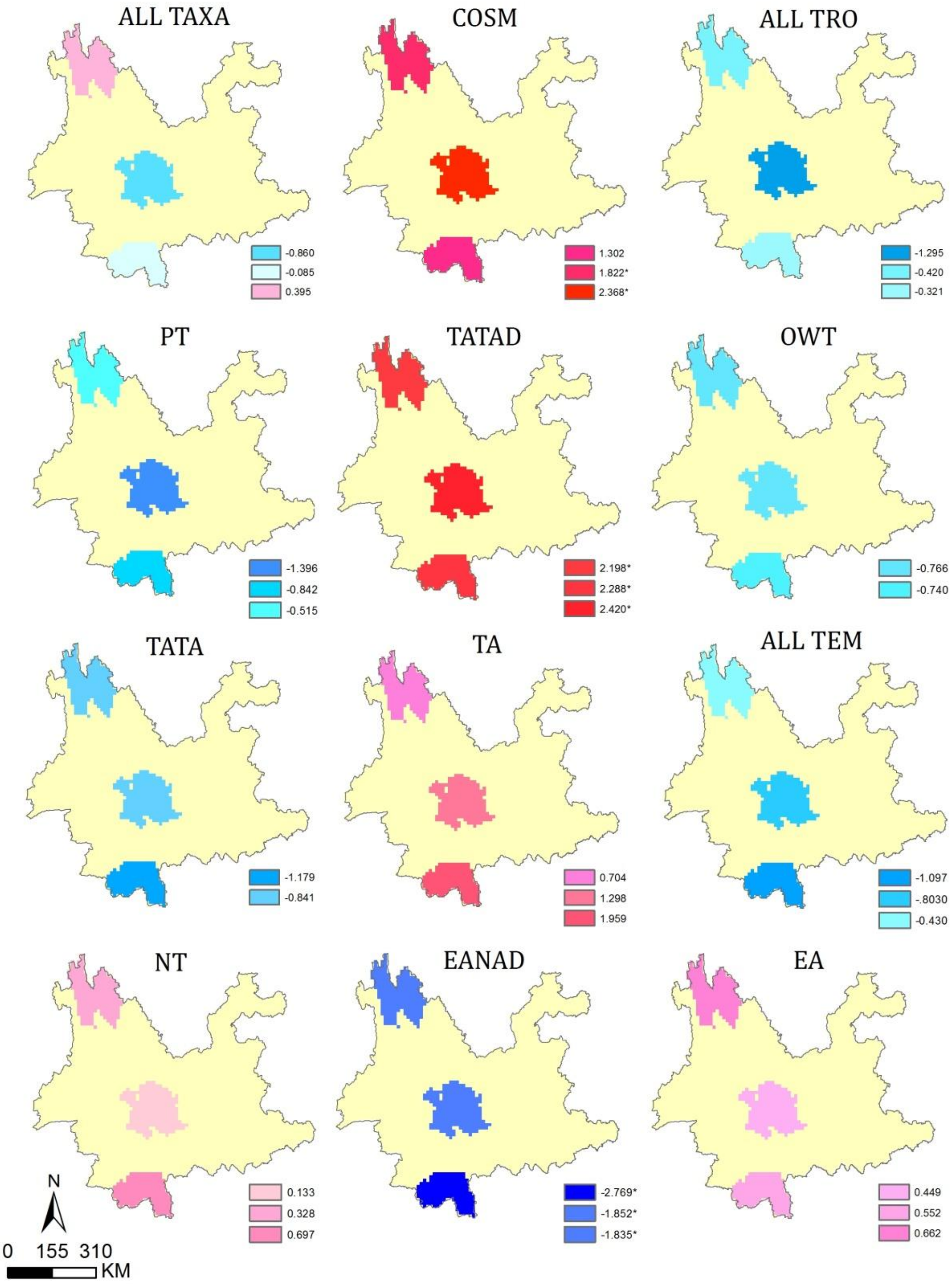

*The *p*-values corresponding to *NRI* less than 0.05.

ALL TAXA, all taxa at the family level; COSM, cosmopolitan; ALL TRO, all taxa with tropical distributions; PT, pantropic; TATAD, tropical Asia and tropical America disjointed; OWT, old world tropic; TATA, tropical Asia to tropical Australia; TA, tropical Asia; ALL TEM, all taxa with temperate distributions; NT, north temperate; EANAD, east Asia and north America disjointed; and EA, east Asia.

Figure S2. The nearest taxon index (*NTI*) of the three floras at the family level.

The map was generated using ArcGIS 10.2.2. (http://www.esri.com)

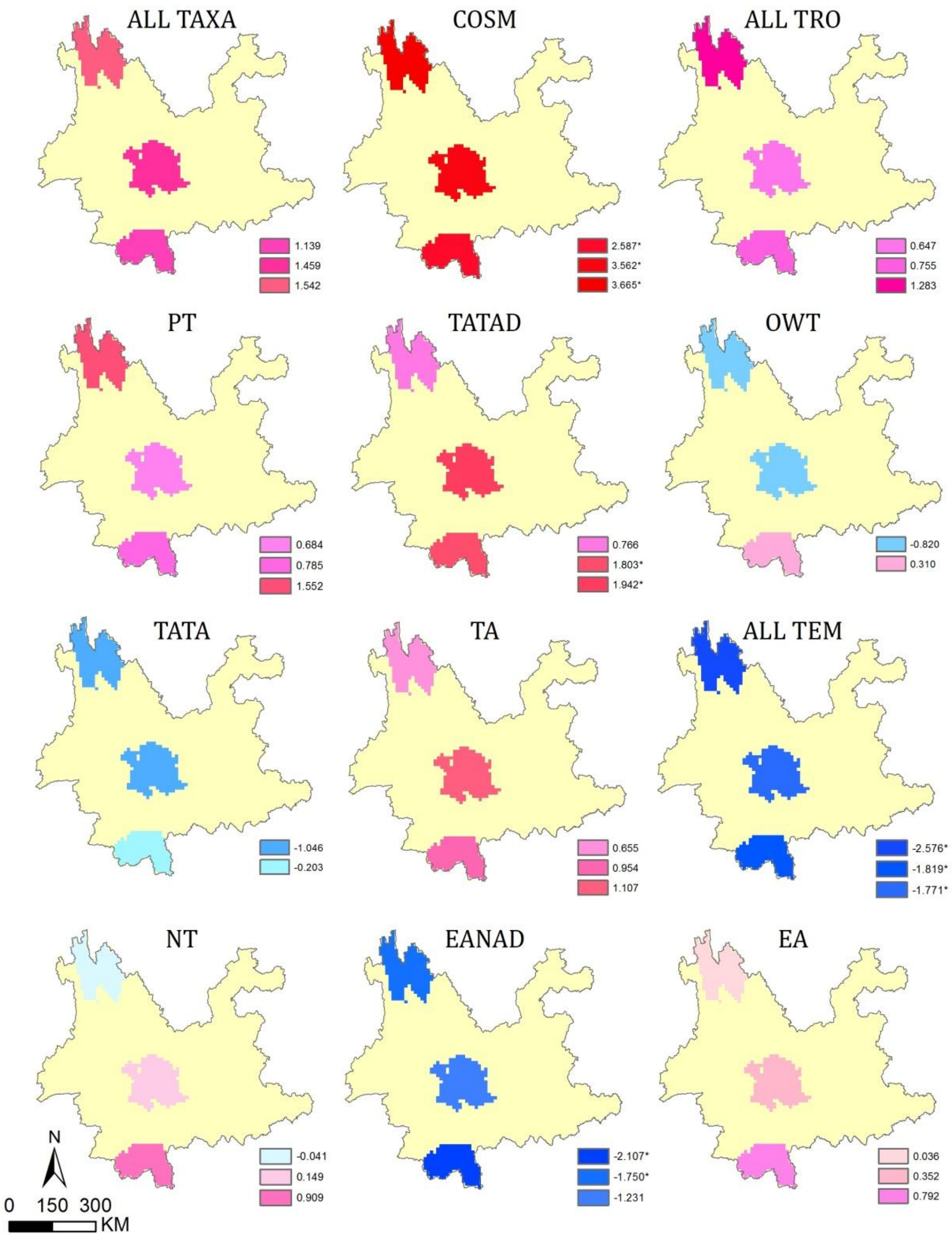

*The *p*-values corresponding to *NTI* less than 0.05.

Figure S3. The Phylogenetic tree constructed from S.PhyloMaker of 212 families in three representative regions across Yunnan.

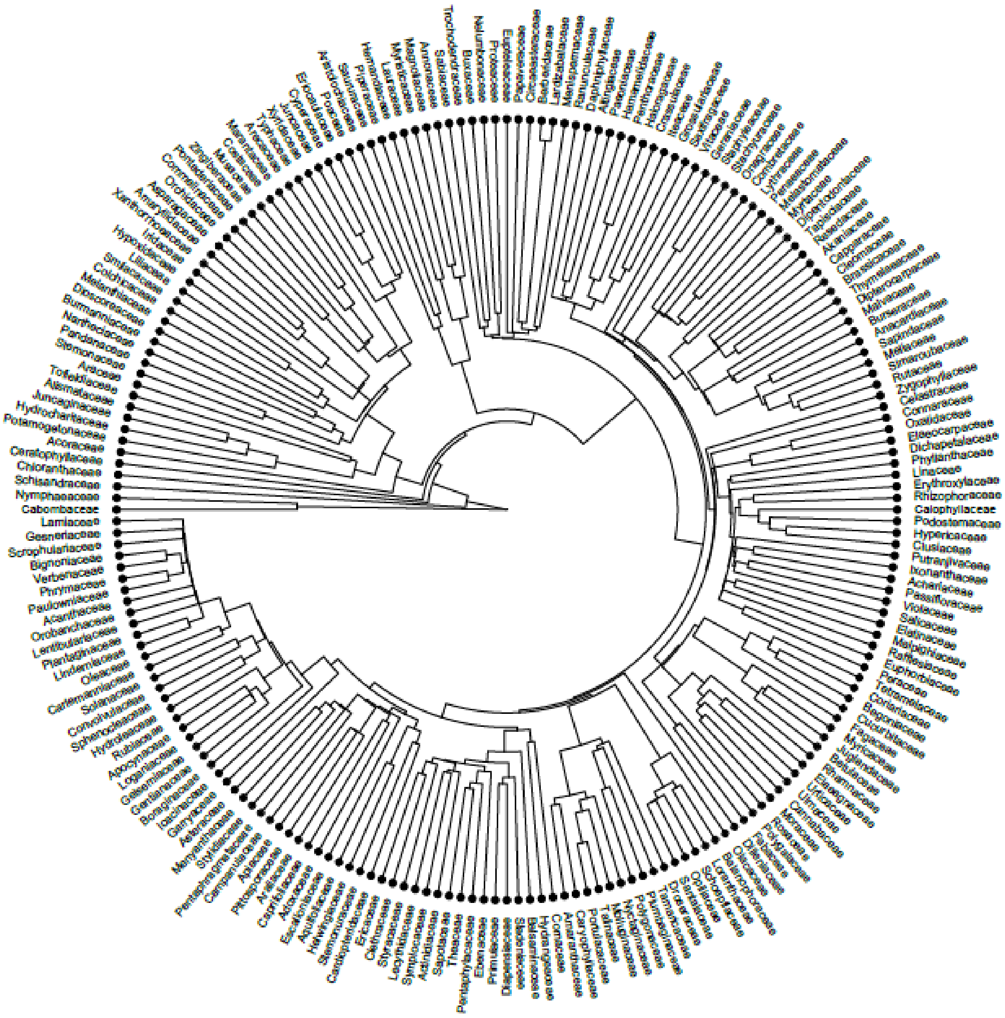

Figure S4. The Phylogenetic tree of 1,860 genera in three representative regions across Yunnan, constructed from S.PhyloMaker with adding missing genera as polytomies within their families.

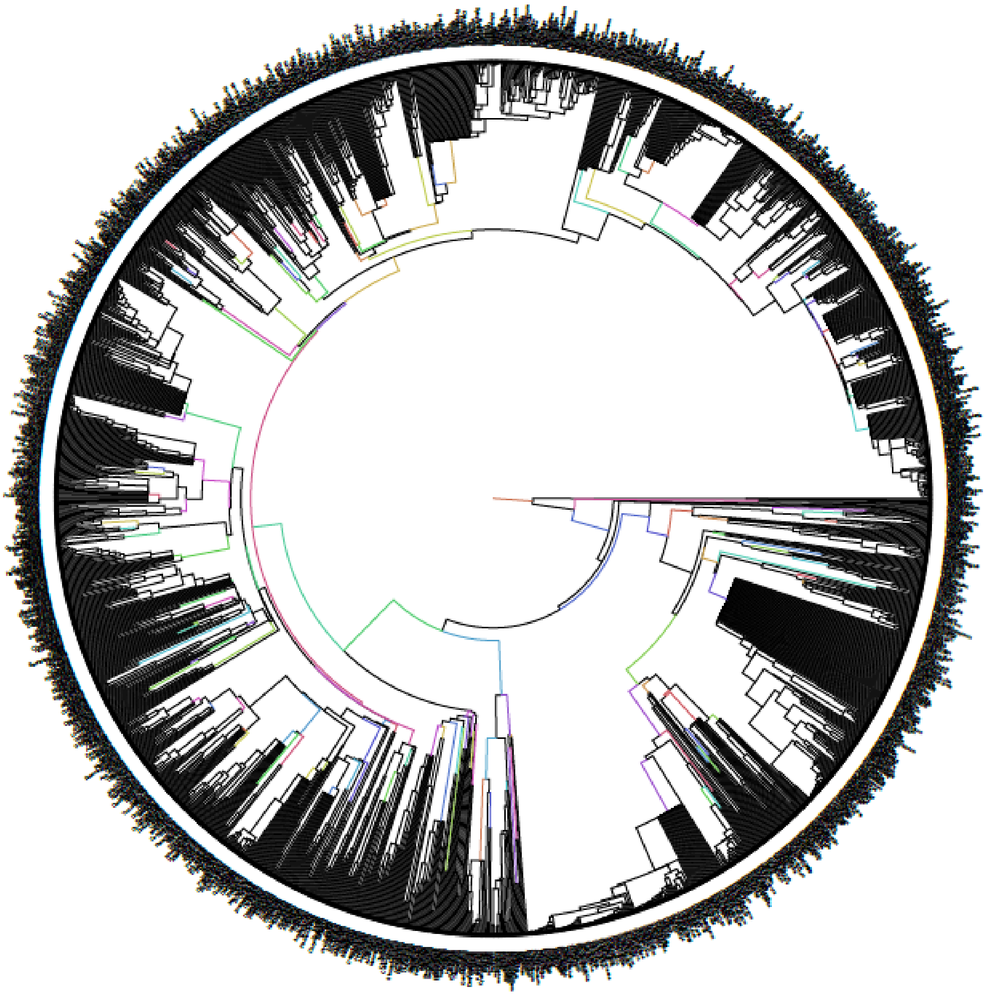

Figure S5. The Phylogenetic tree of 9,370 species in three representative regions across Yunnan, constructed from S.PhyloMaker with adding missing species as polytomies within their genera.

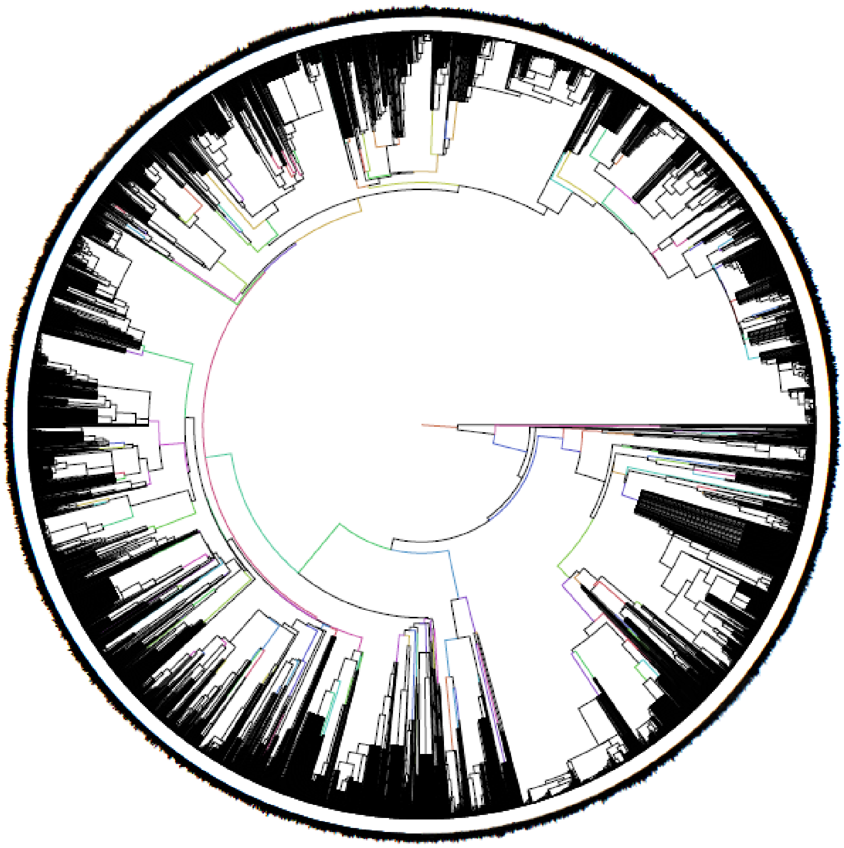

Tables

Table S1 Detailed results of phylogenetic structure (including all *p*-values corresponding to *NRI* and *NTI*) of the three representative floras across Yunnan according to the first approach which adds missing genera or species as polytomies within their families or genera.

	Flora of southern Yunnan			Flora of central Yunnan			Flora of northwestern Yunnan		
	TR	*NRI(p)*	*NTI(p)*	*TR*	*NRI(p)*	*NTI(p)*	*TR*	*NRI(p)*	*NTI(p)*
At the family level									
All families	193	-0.085 (0.53)	1.139 (0.14)	174	-0.860 (0.80)	1.459 (0.09)	170	0.395 (0.32)	1.542 (0.06)
Cosmopolitan	51	1.302 (0.11)	2.587 (0.01)	50	2.368 (0.01)	3.562 (0.01)	53	1.822 (0.05)	3.665 (0.01)
Tropical distributions	104	-0.321 (0.62)	0.755 (0.22)	87	-1.295 (0.92)	0.647 (0.28)	75	-0.420 (0.65)	1.283 (0.12)
PT	72	-0.842 (0.78)	0.785 (0.23)	63	-1.396 (0.94)	0.684 (0.27)	54	-0.515 (0.68)	1.552 (0.07)
TATAD	11	2.288 (0.02)	1.803 (0.05)	11	2.420 (0.01)	1.942 (0.04)	11	2.198 (0.03)	0.766 (0.26)
OWT	4	-0.740 (0.77)	0.310 (0.41)	3	-0.766 (0.83)	-0.820 (0.83)	3	-0.766 (0.83)	-0.820 (0.83)
TATA	5	-1.179 (0.92)	-0.203 (0.60)	4	-0.841 (0.82)	-1.046 (0.87)	4	-0.841 (0.82)	-1.046 (0.87)
TATAF	3	-0.640 (0.61)	-0.304 (0.63)	1	—	—	0	—	—
TA	9	1.959 (0.06)	0.954 (0.17)	5	1.298 (0.17)	1.107 (0.14)	3	0.704 (0.37)	0.655 (0.25)
Temperate distributions	31	-1.097 (0.89)	-1.819 (0.97)	34	-0.803 (0.80)	-1.771 (0.96)	40	-0.430 (0.67)	-2.576 (0.99)
NT	19	0.697 (0.24)	0.909 (0.20)	23	0.133 (0.43)	0.149 (0.47)	25	0.328 (0.35)	-0.041 (0.57)
EANAD	10	-2.769 (1.00)	-2.107 (0.97)	8	-1.835 (0.97)	-1.231 (0.89)	9	-1.852 (0.99)	-1.750 (0.98)
OWTE	0	—	—	0	—	—	1	—	—
EA	2	0.552 (0.41)	0.792 (0.29)	3	0.449 (0.44)	0.352 (0.39)	5	0.662 (0.27)	0.036 (0.44)
EC	1	—	—	1	—	—	1	—	—
ESHD	1	—	—	0	—	—	0	—	—
At the genus level									

All genera	1238	-3.178 (1.00)	-0.061 (0.52)	1056	0.725 (0.23)	2.041 (0.02)	1250	3.225 (0.00)	2.472 (0.01)
Cosmopolitan	65	-2.241 (0.99)	1.555 (0.07)	67	-0.163 (0.55)	2.163 (0.02)	83	-0.823 (0.79)	2.677 (0.01)
Tropical distributions	949	-3.017 (1.00)	1.518 (0.07)	643	1.327 (0.10)	2.910 (0.00)	544	-1.727 (0.96)	2.976 (0.00)
PT	261	-0.169 (0.55)	1.801 (0.03)	205	0.868 (0.20)	1.845 (0.04)	190	-0.255 (0.58)	2.705 (0.00)
TATAD	31	0.250 (0.37)	0.837 (0.21)	36	2.442 (0.02)	0.706 (0.24)	30	2.384 (0.02)	1.151 (0.13)
OWT	130	-0.261 (0.58)	3.586 (0.00)	95	0.496 (0.30)	4.012 (0.00)	79	-0.182 (0.55)	3.761 (0.00)
TATA	135	-2.766 (1.00)	0.736 (0.22)	82	-1.012 (0.85)	1.148 (0.12)	62	-2.014 (0.99)	-0.453 (0.67)
TATAF	70	3.103 (0.01)	1.695 (0.04)	52	3.165 (0.00)	1.542 (0.06)	45	0.839 (0.19)	1.165 (0.13)
TA	322	-1.738 (0.97)	-0.077 (0.54)	173	0.055 (0.46)	1.301 (0.09)	138	-0.639 (0.72)	1.999 (0.02)
Temperate distributions	211	2.096 (0.03)	-0.339 (0.64)	331	3.284 (0.00)	1.488 (0.07)	573	3.141 (0.00)	1.065 (0.14)
NT	71	1.300 (0.10)	-0.076 (0.52)	130	1.205 (0.11)	0.630 (0.24)	207	0.059 (0.45)	0.547 (0.30)
EANAD	33	0.771 (0.21)	-0.587 (0.72)	38	0.662 (0.25)	0.193 (0.42)	65	0.455 (0.32)	-0.197 (0.59)
OWTE	27	1.932 (0.04)	1.505 (0.07)	45	2.713 (0.01)	3.801 (0.00)	90	3.985 (0.00)	3.657 (0.00)
TEA	6	0.846 (0.19)	1.840 (0.05)	6	0.824 (0.18)	1.632 (0.07)	16	1.412 (0.09)	2.806 (0.00)
MWACA	5	0.026 (0.41)	-0.150 (0.50)	7	0.463 (0.33)	-0.574 (0.69)	17	0.613 (0.26)	1.850 (0.04)
CA	2	-1.049 (0.62)	-1.098 (0.63)	2	1.018 (0.10)	0.938 (0.09)	12	1.672 (0.06)	0.675 (0.25)
EA	67	0.768 (0.21)	0.552 (0.28)	103	1.757 (0.05)	-0.653 (0.74)	166	1.809 (0.05)	-0.280 (0.62)
EC	13	2.284 (0.03)	-0.037 (0.52)	14	0.506 (0.27)	-0.430 (0.64)	46	2.350 (0.02)	0.533 (0.30)
At the species level									
All species	3785	-9.177 (1.00)	2.136 (0.03)	2972	4.816 (0.01)	6.545 (0.01)	6107	6.625 (0.01)	5.809 (0.01)

TR, taxon richness; *NRI*, net relatedness index; *NTI*, nearest taxon index; PT, pantropic; TATAD, tropical Asia and tropical America disjointed; OWT, old world tropic; TATA, tropical Asia to tropical Australia; TATAF, tropical Asia to tropical

Africa; TA, tropical Asia; NT, north temperate; EANAD, east Asia and north America disjointed; OWTE, old world temperate; TEA, temperate Asia; MWACA, Mediterranean, west Asia to center Asia; CA, center Asia; EA, east Asia; EC, endemic to China; and ESHD, extratropical southern hemisphere disjointed or dispersed.

Table S2 Detailed results of phylogenetic analyses on three representative floras across Yunnan according to the second approach which randomly adds missing genera or species within their families or genera.

(a) Phylogenetic structure of the three floras at the family, genus and species levels.

	Flora of southern Yunnan			Flora of central Yunnan			Flora of northwestern Yunnan		
	TR	*NRI (p)*	*NTI (p)*	*TR*	*NRI (p)*	*NTI (p)*	*TR*	*NRI (p)*	*NTI (p)*
At the family level									
All families	193	-0.136 (0.56)	1.207 (0.14)	174	-0.794 (0.78)	1.492 (0.07)	170	0.524 (0.31)	1.483 (0.09)
Cosmopolitan	51	1.500 (0.06)	2.539 (0.02)	50	2.645 (0.01)	3.595 (0.01)	53	2.091 (0.02)	3.646 (0.01)
Tropical distributions	104	-0.293 (0.62)	0.763 (0.20)	87	-1.537 (0.95)	0.592 (0.27)	75	-0.409 (0.64)	1.112 (0.12)
PT	72	-0.939 (0.86)	0.857 (0.16)	63	-1.616 (0.95)	0.690 (0.27)	54	-0.474 (0.70)	1.427 (0.06)
TATAD	11	1.791 (0.06)	1.784 (0.04)	11	2.067 (0.03)	2.113 (0.02)	11	1.846 (0.06)	0.855 (0.20)
OWT	4	-0.788 (0.77)	0.295 (0.36)	3	-0.791 (0.83)	-0.941 (0.85)	3	-0.791 (0.83)	-0.941 (0.85)
TATA	5	-0.884 (0.84)	-0.383 (0.61)	4	-0.636 (0.75)	-1.292 (0.91)	4	-0.636 (0.75)	-1.292 (0.91)
TATAF	3	-0.670 (0.63)	-0.420 (0.61)	1	—	—	0	—	—
TA	9	2.133 (0.02)	1.168 (0.14)	5	1.331 (0.14)	1.152 (0.10)	3	0.773 (0.32)	0.746 (0.20)
Temperate distributions	31	-1.248 (0.88)	-1.792 (0.97)	34	-0.936 (0.84)	-1.858 (0.96)	40	-0.561 (0.70)	-2.657 (1.00)
NT	19	0.547 (0.33)	0.791 (0.22)	23	0.027 (0.48)	0.038 (0.52)	25	0.229 (0.40)	-0.154 (0.59)
EANAD	10	-2.624 (1.00)	-2.182 (1.00)	8	-1.796 (0.96)	-1.263 (0.91)	9	-1.792 (0.96)	-1.719 (0.97)
OWTE	0	—	—	0	—	—	1	—	—
EA	2	0.738 (0.35)	0.698 (0.34)	3	0.606 (0.36)	0.370 (0.43)	5	0.795 (0.22)	0.051 (0.45)
EC	1	—	—	1	—	—	1	—	—
ESHD	1	—	—	0	—	—	0	—	—
At the genus level									

All genera	1238	-3.185 (1.00)	-0.016 (0.51)	1056	3.036 (0.00)	1.586 (0.05)	1250	0.803 (0.21)	2.449 (0.01)
Cosmopolitan	65	-2.220 (0.99)	1.898 (0.02)	67	-0.202 (0.56)	2.633 (0.01)	83	-0.851 (0.81)	3.184 (0.00)
Tropical distributions	949	-3.000 (1.00)	2.873 (0.00)	643	1.251 (0.11)	2.777 (0.00)	544	-1.654 (0.95)	2.983 (0.00)
PT	261	-0.234 (0.57)	1.747 (0.03)	205	0.860 (0.20)	2.001 (0.02)	190	-0.343 (0.61)	2.591 (0.00)
TATAD	31	0.243 (0.38)	0.062 (0.50)	36	2.539 (0.01)	0.191 (0.44)	30	2.422 (0.02)	0.517 (0.31)
OWT	130	-0.338 (0.60)	3.354 (0.00)	95	0.473 (0.31)	3.766 (0.00)	79	-0.228 (0.56)	3.502 (0.00)
TATA	135	-2.433 (1.00)	1.975 (0.03)	82	-0.755 (0.76)	1.517 (0.06)	62	-1.603 (0.95)	0.09 (0.46)
TATAF	70	3.034 (0.00)	1.421 (0.07)	52	3.201 (0.01)	1.215 (0.11)	45	0.881 (0.19)	0.642 (0.26)
TA	322	-1.561 (0.94)	2.060 (0.02)	173	0.056 (0.46)	2.073 (0.02)	138	-0.573 (0.71)	2.270 (0.02)
Temperate distributions	211	2.116 (0.02)	-0.299 (0.62)	331	3.239 (0.00)	2.066 (0.02)	573	3.326 (0.00)	2.706 (0.01)
NT	71	1.361 (0.10)	0.282 (0.39)	130	1.185 (0.13)	1.020 (0.16)	207	0.190 (0.41)	2.093 (0.02)
EANAD	33	0.753 (0.21)	-0.997 (0.85)	38	0.671 (0.25)	-0.013 (0.51)	65	0.363 (0.34)	-0.228 (0.60)
OWTE	27	1.949 (0.04)	1.227 (0.11)	45	2.801 (0.01)	3.776 (0.00)	90	4.094 (0.00)	4.023 (0.00)
TEA	6	0.884 (0.18)	1.636 (0.06)	6	0.807 (0.17)	1.550 (0.08)	16	1.395 (0.10)	2.536 (0.01)
MWACA	5	0.064 (0.36)	-0.253 (0.56)	7	0.457 (0.28)	-0.617 (0.70)	17	0.693 (0.24)	1.858 (0.04)
CA	2	-1.042 (0.62)	-1.121 (0.65)	2	0.825 (0.12)	1.001 (0.10)	12	1.625 (0.07)	0.690 (0.25)
EA	67	0.842 (0.21)	1.290 (0.10)	103	1.953 (0.03)	0.348 (0.38)	166	1.901 (0.03)	0.951 (0.17)
EC	13	2.220 (0.03)	0.402 (0.32)	14	0.466 (0.30)	-0.645 (0.74)	46	2.386 (0.01)	0.977 (0.17)
At the species level									
All species	3785	-8.882 (1.00)	-4.238 (1.00)	2972	4.590 (0.01)	0.680 (0.28)	6107	6.496 (0.01)	8.580 (0.01)

TR, taxon richness; *NRI*, net relatedness index; *NTI*, nearest taxon index; PT, pantropic; TATAD, tropical Asia and tropical America disjointed; OWT, old world tropic; TATA, tropical Asia to tropical Australia; TATAF, tropical Asia to tropical

Africa; TA, tropical Asia; NT, north temperate; EANAD, east Asia and north America disjointed; OWTE, old world temperate; TEA, temperate Asia; MWACA, Mediterranean, west Asia to center Asia; CA, center Asia EA, east Asia; EC, endemic to China; and ESHD, extratropical southern hemisphere disjointed or dispersed.

(b) Phylogenetic similarity (*PhyloSor*) among the three floras at the family, genus and species levels.

	At the family level			At the genus level			At the species level		
	BN-ZB	ZB-DQ	DQ-BN	BN-ZB	ZB-DQ	DQ-BN	BN-ZB	ZB-DQ	DQ-BN
All taxa	0.904	0.906	0.837	0.785	0.782	0.676	0.649	0.626	0.490
Cosmopolitan	0.944	0.976	0.955	0.909	0.886	0.852	—	—	—
tropical distributions	0.918	0.888	0.828	0.788	0.820	0.710	—	—	—
PT	0.948	0.876	0.852	0.828	0.878	0.794	—	—	—
TATAD	0.925	0.939	0.867	0.795	0.874	0.766	—	—	—
OWT	0.913	1.000	0.913	0.840	0.806	0.768	—	—	—
TATA	0.923	1.000	0.923	0.786	0.809	0.702	—	—	—
TATAF	0.614	—	—	0.770	0.852	0.713	—	—	—
TA	0.766	0.827	0.609	0.715	0.726	0.601	—	—	—
Temperate distributions	0.880	0.903	0.813	0.781	0.766	0.639	—	—	—
NT	0.899	0.962	0.862	0.752	0.842	0.653	—	—	—
EANAD	0.880	0.885	0.851	0.810	0.683	0.627	—	—	—
OWTE	—	—	—	0.729	0.684	0.573	—	—	—
TEA	—	—	—	0.813	0.507	0.410	—	—	—
MWACA	—	—	—	0.870	0.780	0.660	—	—	—
CA	—	—	—	0.380	0.365	0.401	—	—	—
EA	0.841	0.801	0.653	0.732	0.803	0.605	—	—	—
EC	1.000	0.437	0.437	0.553	0.445	0.285	—	—	—
ESHD	—	—	—	—	—	—	—	—	—

BN, southern Yunnan; ZB, central Yunnan; and DQ, northwestern Yunnan.

(c) The standardized effect sizes of phylogenetic beta diversity (*S.E.S.* D_{nn}) among the three floras at the family, genus and species levels.

Groups	At the family level			At the genus level			At the species level		
	BN-ZB	ZB-DQ	DQ-BN	BN-ZB	ZB-DQ	DQ-BN	BN-ZB	ZB-DQ	DQ-BN
All taxa	138.45	107.22	161.16	242.34	241.11	354.40	329.58	444.56	638.83
Cosmopolitan	25.41	26.58	25.95	29.92	32.20	31.89	—	—	—
tropical distributions	41.18	34.96	36.98	147.97	119.83	132.17	—	—	—
PT	31.66	26.67	27.71	59.60	55.04	56.11	—	—	—
TATAD	9.81	10.21	9.92	18.48	19.89	17.79	—	—	—
OWT	4.76	4.75	4.76	38.74	36.37	37.36	—	—	—
TATA	5.93	5.97	5.93	37.53	32.12	33.45	—	—	—
TATAF	0.23	—	—	27.65	24.48	25.86	—	—	—
TA	6.81	5.01	4.63	58.45	47.83	54.24	—	—	—
Temperate distributions	19.04	21.00	19.13	68.68	92.71	79.32	—	—	—
NT	13.99	15.97	14.22	36.60	50.77	39.55	—	—	—
EANAD	9.34	9.16	9.50	19.78	21.37	19.59	—	—	—
OWTE	—	—	—	19.30	25.95	19.38	—	—	—
TEA	—	—	—	7.47	7.71	6.15	—	—	—
MWACA	—	—	—	6.56	10.04	6.62	—	—	—
CA	—	—	—	0.77	3.52	2.85	—	—	—
EA	3.58	4.67	3.24	30.53	40.55	31.67	—	—	—
EC	—	0.21	0.21	8.71	12.38	8.95	—	—	—
ESHD	—	—	—	—	—	—	—	—	—

BN, southern Yunnan; ZB, central Yunnan; and DQ, northwestern Yunnan.

Table S3 Detailed results of phylogenetic analyses on three representative floras across Yunnan according to the third approach which adds missing genera or species to their families or genera with the same approach used in Phylomatic and BLADJ.

(a) Phylogenetic structure of the three floras at the family, genus and species levels.

	Flora of southern Yunnan			Flora of central Yunnan			Flora of northwestern Yunnan		
	TR	*NRI (p)*	*NTI (p)*	*TR*	*NRI (p)*	*NTI (p)*	*TR*	*NRI (p)*	*NTI (p)*
At the family level									
All families	193	-0.086 (0.51)	1.225 (0.12)	174	-0.797 (0.79)	1.524 (0.06)	170	0.414 (0.32)	1.585 (0.06)
Cosmopolitan	51	1.381 (0.10)	2.711 (0.01)	50	2.320 (0.02)	3.693 (0.01)	53	1.805 (0.04)	3.884 (0.01)
Tropical distributions	104	-0.345 (0.63)	0.753 (0.21)	87	-1.445 (0.93)	0.691 (0.26)	75	-0.401 (0.66)	1.219 (0.11)
PT	72	-0.948 (0.82)	0.828 (0.21)	63	-1.513 (0.95)	0.777 (0.21)	54	-0.515 (0.73)	1.578 (0.06)
TATAD	11	2.275 (0.01)	1.675 (0.05)	11	2.350 (0.03)	1.975 (0.03)	11	2.105 (0.03)	0.843 (0.22)
OWT	4	-0.635 (0.73)	0.190 (0.41)	3	-0.727 (0.78)	-0.957 (0.81)	3	-0.727 (0.78)	-0.957 (0.81)
TATA	5	-0.968 (0.88)	-0.217 (0.59)	4	-0.707 (0.78)	-1.258 (0.89)	4	-0.707 (0.78)	-1.258 (0.89)
TATAF	3	-0.694 (0.58)	-0.366 (0.59)	1	—	—	0	—	—
TA	9	1.997 (0.05)	1.160 (0.14)	5	1.261 (0.18)	1.008 (0.14)	3	0.693 (0.36)	0.562 (0.27)
Temperate distributions	31	-1.108 (0.89)	-1.795 (0.98)	34	-0.760 (0.80)	-1.714 (0.96)	40	-0.454 (0.67)	-2.495 (0.99)
NT	19	0.633 (0.22)	0.870 (0.19)	23	0.141 (0.44)	0.147 (0.45)	25	0.343 (0.34)	-0.066 (0.52)
EANAD	10	-2.608 (1.00)	-2.179 (0.99)	8	-1.676 (0.95)	-1.193 (0.90)	9	-1.761 (0.97)	-1.685 (0.97)
OWTE	0	—	—	0	—	—	1	—	—
EA	2	0.781 (0.32)	0.733 (0.35)	3	0.554 (0.40)	0.391 (0.40)	5	0.789 (0.24)	0.181 (0.38)
EC	1	—	—	1	—	—	1	—	—

ESHD	1	—	—	0	—	—	0	—	—
At the genus level									
All genera	1238	-3.282 (1.00)	1.679 (0.05)	1056	3.273 (0.00)	5.334 (0.00)	1250	0.913 (0.19)	4.597 (0.00)
Cosmopolitan	65	-2.195 (0.99)	3.248 (0.00)	67	-0.120 (0.52)	4.030 (0.00)	83	-0.764 (0.77)	4.872 (0.00)
Tropical distributions	949	-3.015 (1.00)	1.575 (0.05)	643	1.334 (0.10)	3.751 (0.00)	544	-1.722 (0.96)	3.694 (0.00)
PT	261	-0.178 (0.57)	3.750 (0.00)	205	0.851 (0.21)	3.474 (0.00)	190	-0.335 (0.63)	4.057 (0.00)
TATAD	31	0.170 (0.39)	-0.769 (0.78)	36	2.368 (0.02)	0.251 (0.40)	30	2.288 (0.02)	0.557 (0.28)
OWT	130	-0.250 (0.60)	3.916 (0.00)	95	0.573 (0.28)	4.363 (0.00)	79	-0.152 (0.53)	3.833 (0.00)
TATA	135	-2.758 (1.00)	1.346 (0.10)	82	-0.902 (0.82)	1.284 (0.10)	62	-1.907 (0.98)	-0.268 (0.60)
TATAF	70	3.175 (0.00)	1.959 (0.03)	52	3.060 (0.01)	1.828 (0.03)	45	0.912 (0.19)	1.275 (0.11)
TA	322	-1.698 (0.96)	-1.585 (0.95)	173	0.070 (0.45)	0.512 (0.31)	138	-0.733 (0.75)	1.263 (0.10)
Temperate distributions	71	1.991 (0.03)	1.484 (0.06)	130	3.282 (0.00)	4.430 (0.00)	207	3.215 (0.00)	3.817 (0.00)
NT	211	1.354 (0.09)	1.753 (0.04)	331	1.285 (0.10)	2.856 (0.00)	573	0.279 (0.40)	4.227 (0.00)
EANAD	33	0.818 (0.21)	-0.520 (0.70)	38	0.709 (0.24)	0.524 (0.30)	65	0.403 (0.32)	0.914 (0.18)
OWTE	27	1.996 (0.04)	1.910 (0.03)	45	2.859 (0.00)	4.656 (0.00)	90	4.064 (0.00)	5.253 (0.00)
TEA	6	0.822 (0.19)	1.880 (0.05)	6	0.735 (0.20)	1.718 (0.06)	16	1.370 (0.10)	2.387 (0.01)
MWACA	5	0.053 (0.38)	-0.121 (0.50)	7	0.513 (0.28)	-0.497 (0.65)	17	0.524 (0.27)	2.111 (0.02)
CA	2	-1.207 (0.67)	-1.035 (0.60)	2	0.948 (0.11)	0.879 (0.11)	12	1.354 (0.11)	-0.008 (0.49)
EA	67	0.758 (0.22)	1.562 (0.06)	103	1.827 (0.04)	0.056 (0.48)	166	1.705 (0.06)	-0.803 (0.80)
EC	13	2.229 (0.03)	0.012 (0.48)	14	0.493 (0.30)	-0.417 (0.66)	46	2.230 (0.02)	-0.502 (0.71)
At the species level									
All species	3785	-10.377 (1.00)	2.370 (0.01)	2972	4.887 (0.01)	6.784 (0.01)	6107	7.230 (0.01)	6.119 (0.01)

TR, taxon richness; *NRI*, net relatedness index; *NTI*, nearest taxon index; PT, pantropic; TATAD, tropical Asia and tropical America disjointed; OWT, old world

tropic; TATA, tropical Asia to tropical Australia; TATAF, tropical Asia to tropical Africa; TA, tropical Asia; NT, north temperate; EANAD, east Asia and north America disjointed; OWTE, old world temperate; TEA, temperate Asia; MWACA, Mediterranean, west Asia to center Asia; CA, center Asia; EA, east Asia; EC, endemic to China; and ESHD, extratropical southern hemisphere disjointed or dispersed.

(b) Phylogenetic similarity (*PhyloSor*) among the three floras at the family, genus and species levels.

	At the family level			At the genus level			At the species level		
	BN-ZB	ZB-DQ	DQ-BN	BN-ZB	ZB-DQ	DQ-BN	BN-ZB	ZB-DQ	DQ-BN
All taxa	0.904	0.906	0.837	0.734	0.727	0.595	0.528	0.449	0.322
Cosmopolitan	0.944	0.976	0.955	0.907	0.885	0.851	—	—	
tropical distributions	0.918	0.888	0.828	0.733	0.785	0.641	—	—	—
PT	0.948	0.876	0.852	0.818	0.856	0.763	—	—	—
TATAD	0.925	0.939	0.867	0.780	0.872	0.754	—	—	
OWT	0.913	1.000	0.913	0.822	0.786	0.754	—	—	—
TATA	0.923	1.000	0.923	0.769	0.789	0.683	—	—	—
TATAF	0.614	0.000	0.000	0.759	0.841	0.696	—	—	
TA	0.766	0.827	0.609	0.645	0.700	0.509	—	—	—
Temperate distributions	0.880	0.903	0.813	0.749	0.710	0.574	—	—	—
NT	0.899	0.962	0.861	0.736	0.822	0.624	—	—	
EANAD	0.880	0.885	0.851	0.808	0.679	0.622	—	—	
OWTE	—	—	—	0.714	0.652	0.549	—	—	—
TEA	—	—	—	0.813	0.490	0.396	—	—	—
MWACA	—	—	—	0.870	0.761	0.642	—	—	
CA	—	—	—	0.380	0.342	0.377	—	—	
EA	0.841	0.801	0.653	0.703	0.745	0.543	—	—	—
EC	1.000	0.437	0.437	0.543	0.403	0.258	—	—	—
ESHD	—	—	—	—	—	—	—	—	

BN, southern Yunnan; ZB, central Yunnan; and DQ, northwestern Yunnan.

(c) The standardized effect sizes of phylogenetic beta diversity (*S.E.S.* D_{nn}) among the three floras at the family, genus and species levels.

Groups	At the family level			At the genus level			At the species level		
	BN-ZB	ZB-DQ	DQ-BN	BN-ZB	ZB-DQ	DQ-BN	BN-ZB	ZB-DQ	DQ-BN
All taxa	141.03	102.85	162.82	236.10	224.68	350.09	304.50	410.84	589.18
Cosmopolitan	26.87	27.85	27.50	29.55	30.03	31.01	—	—	—
tropical distributions	38.13	33.61	34.90	129.85	111.33	127.08	—	—	—
PT	30.82	26.80	28.08	59.69	52.28	57.27	—	—	—
TATAD	10.62	10.91	10.29	18.94	19.22	17.62	—	—	—
OWT	4.98	4.92	4.98	36.89	31.73	33.05	—	—	—
TATA	6.09	6.13	6.09	34.80	29.25	31.46	—	—	—
TATAF	0.12	—	—	26.79	23.66	25.76	—	—	—
TA	6.71	5.20	4.62	53.63	46.82	48.42	—	—	—
Temperate distributions	16.45	18.51	16.89	59.22	82.53	64.52	—	—	—
NT	13.43	15.02	13.58	29.93	47.19	32.40	—	—	—
EANAD	7.79	7.56	7.72	17.73	19.27	17.43	—	—	—
OWTE	—	—	—	19.75	27.72	19.35	—	—	—
TEA	—	—	—	7.69	7.89	5.90	—	—	—
MWACA	—	—	—	6.86	9.80	6.91	—	—	—
CA	—	—	—	0.88	3.39	2.98	—	—	—
EA	3.48	4.68	3.20	29.78	40.29	30.71	—	—	—
EC	—	0.16	0.16	8.98	11.05	8.23	—	—	—
ESHD	—	—	—	—	—	—	—	—	—

BN, southern Yunnan; ZB, central Yunnan; and DQ, northwestern Yunnan.
